# Acute myocardial infarctions and stroke triggered by laboratory-confirmed respiratory infections in Denmark, 2010 to 2016

**DOI:** 10.2807/1560-7917.ES.2020.25.17.1900199

**Published:** 2020-04-30

**Authors:** Jessica Ohland, Charlotte Warren-Gash, Ruth Blackburn, Kåre Mølbak, Palle Valentiner-Branth, Jens Nielsen, Hanne-Dorthe Emborg

**Affiliations:** 1 Statens Serum Institut, Copenhagen, Denmark; 2 Faculty of Epidemiology & Population Health, London School of Hygiene & Tropical Medicine, London, United Kingdom; 3 Institute of Health Informatics, University College London, London, United Kingdom; 4 Institute of Veterinary and Animal Sciences, Faculty of Health and Medical Sciences, University of Copenhagen, Denmark

**Keywords:** acute myocardial infarction, ischemic stroke, respiratory infections self-controlled case series method

## Abstract

**Background:**

Several studies have investigated a possible association between respiratory infection and acute myocardial infarction (MI). As both influenza and pneumococcal infections are vaccine preventable, understanding the populations affected by virus-induced cardiovascular complications is important to guide public health and clinical practice.

**Aim:**

This observational study aimed to quantify the association between laboratory-confirmed respiratory bacteria or virus infections and risk of first MI or stroke, by using self-controlled case series (SCCS) analysis of anonymised linked electronic Danish health records.

**Methods:**

The SCCS method was used to determine the relative incidence of the first event of MI and stroke occurring within 28 days after laboratory-confirmed respiratory infections compared with the baseline time period.

**Results:**

In the age and season adjusted analyses for first acute MI, the incidence ratios (IR) of a MI event occurring during the risk period were significantly elevated following a *Streptococcus pneumoniae* infection with values of 20.1, 11.0 and 4.9 during 1–3, 4–7 and 8–14 days, respectively and following respiratory virus infection with values of 15.2, 4.5 and 4.4 during 1–3, 8–14 and 15–28 days, respectively. The significantly elevated IRs for stroke following an *S. pneumoniae* infection were 25.5 and 6.3 during 1–3 and 8–14 days, respectively and following respiratory virus infection 8.3, 7.8 and 6.2 during 1–3, 4–7 and 8–14 days, respectively.

**Conclusion:**

This study suggested a significant cardiovascular event triggering effect following infection with *S. pneumoniae* and respiratory viruses (mainly influenza), indicating the importance of protection against vaccine-preventable respiratory infections.

## Introduction

The leading causes of death worldwide for the past 15 years were ischaemic heart disease and stroke [1], which is also the case in Denmark (http://www.healthdata.org/denmark). An association between circulation of seasonal influenza and acute cardiovascular events was first suggested in the 1930s, although these findings were limited by risk of ecological bias and lack of control for potential confounding factors [2]. A systematic review from 2009 found that influenza, mostly defined by clinical symptoms alone, appeared to trigger acute myocardial infarction and death from cardiovascular disease [3] and a meta-analysis of case–control studies from 2015 also found a significant association between recent respiratory infection and acute myocardial infarction [4]. Kwong et al. [5] used a laboratory-confirmed influenza virus infection as exposure and found an association with acute myocardial infarction in a Canadian population.

Whether other respiratory organisms might act as triggers for cardiovascular events was investigated in a time series analysis using population-level data from England. This study showed a significant association between the timing of different laboratory-confirmed respiratory viral infections and myocardial infarction and ischaemic stroke hospitalisations among older people. The respiratory viruses investigated included adenovirus, human metapneumovirus, influenza virus, respiratory syncytial virus and rhinovirus [6]. These findings were supported by self-controlled case-series analyses of Scottish individuals, in which incidence ratios (IR) for myocardial infarction and stroke were significantly raised following laboratory-confirmed *Streptococcus pneumoniae* and influenza virus infections, and raised point estimates were also observed following other laboratory-confirmed respiratory virus infections [7]. A systematic review and meta-analysis of observational studies concluded that major cardiac complications occurred in a substantial proportion of the patients with community-acquired pneumonia, which can be caused by a range of pathogens [8]. Currently both influenza virus and pneumococcal infections are vaccine preventable and several vaccines against respiratory syncytial virus are in the pipeline [9]. However influenza and pneumococcal vaccine uptake is sub-optimal across much of Europe. Understanding the relative effects of different organisms on cardiovascular complications as well as the populations affected, will help to inform research into interventions and guide prevention strategies.

Denmark has well-functioning national electronic health registries, including the microbiology database (MiBa) which allows real-time sharing of microbiological test results nationwide and integrates into the national eHealth infrastructure [10]. We aimed to test whether previous findings on cardiovascular events triggering effects of laboratory-confirmed respiratory pathogens also applied to a different northern European population than that of England and Scotland. The aim of our study was to quantify the association between laboratory-confirmed respiratory bacteria or virus infections and risk of first myocardial infarction or stroke using self-controlled case series (SCCS) analysis of anonymised linked electronic health records from Denmark.

## Methods

### Study design

**Figure 1:**
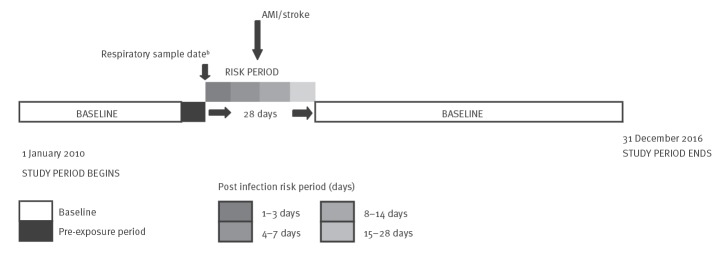
Illustration of the self-controlled case series study method^a^ used in the study, Denmark, 1 January 2010−31 December 2016

We used the SCCS method to estimate the relative incidence of myocardial infarction and stroke following laboratory-confirmed respiratory infections compared with baseline time periods [11] – Figure 1. The SCCS method is best suited for acute events and transient exposures [12]. Only individuals who have a record of exposure (diagnosed with a respiratory infection) and an outcome (myocardial infarction or stroke) were included. The comparisons were made within individuals, implying that factors constant over time cancel out, i.e. that there is implicit control for fixed confounding factors. Factors that varied over time such as age and season were accounted for in the analysis. The study period was 1 January 2010 to 31 December 2016.

AMI: acute myocardial infarction.

^a^ Modified after Petersen et al. [12].

^b^ The date when the sample is taken approximates the date of symptom onset of a respiratory infection.

Following the time of a respiratory infection symptom onset, which was approximated by the date a sample had been taken for laboratory confirmation, a 28 day risk period was considered. Time outside the 28 day window was counted as baseline. The 28 day risk period was further divided into four periods, 1 to 3 days, 4 to 7, 8 to 14 and 15 to 28 days (Figure 1). These periods were chosen for the purpose of direct comparison with other studies. Fourteen days before and the day of sampling were considered as pre-exposure period and excluded from the baseline period, because being diagnosed with an acute myocardial infarction or stroke during this time may change the likelihood of being sampled and diagnosed with a respiratory infection. The SCCS methods compares the likelihood of an acute myocardial infarction or stroke during the risk periods (after a laboratory-confirmed respiratory infection) to the likelihood outside the risk periods (baseline) within a person. Comparing the incidence risk between two time periods gives a relative incidence risk.

### Data sources

#### The Civil Registration System

The Danish Civil Registration System (CRS) was established on 1 April 1968 and since then a unique personal identification number has been assigned to all Danish residents. CRS contains continuously updated information on vital status and permanent residence in Denmark. From CRS, information on birthdate, sex and vital status were extracted.

#### Danish National Patient Register

The Danish National Patient Register (DNPR) contains information on all hospital admissions and outpatient treatments in Denmark [13]. Date of admission and discharge are available for each registration in DNPR. We used date of admission as date of diagnosis for the acute myocardial infarction or stroke. All disease diagnoses are classified according to World Health International Statistical Classification of Diseases and Health-related Problems, 10^th^ Revision (ICD-10*)* [14].

#### Danish Microbiology database

The Danish Microbiology database (MiBa) was initiated in January 2010 and includes all microbiological test results from all the departments of clinical microbiology in Denmark. Each registration contains information on sample date, laboratory analysis and test result [10]. Both records of respiratory virus infections and invasive pneumococcal disease infections were extracted from MiBa. Individuals could be swabbed at a general practitioner’s office, or at a hospital.

#### Pneumococcal laboratory database at Statens Serum Institut 

In Denmark, the national surveillance of invasive pneumococcal disease (IPD) has been centralised at Statens Serum Institut (SSI) since 1938. All clinical microbiology departments in Denmark submit IPD isolates for serotype determination to the National Neisseria and Streptococcus Reference Laboratory (NSR) at SSI.

### Data extraction and linking

All the above registries contain patients’ information under their respective unique personal identification number from CRS, which can be used for record linkage as described below.

Hospital admissions for first acute myocardial infarction (ICD-10 codes: I21 and I23) and/or stroke (ICD-10 codes: I60, I61 and I63) in individuals aged 40 years and above during the study period 1 January 2010 to 31 December 2016 were extracted from the DNPR. In the SCCS method, repeated events must be independent. The risk of a subsequent event may change after experiencing either a myocardial infarction or stoke. We therefore applied a 10 year look back from 1 January 2000 to 31 December 2009 to identify individuals who had hospital admissions for acute myocardial infarction or stroke before the study period.

Specimens testing positive for any of the following viruses from 1 January 2010 to 31 December 2016 were extracted from MiBa: human metapneumovirus, influenza virus, parainfluenza virus, respiratory syncytial virus, or rhinovirus. During the same time period, specimens testing positive for *S. pneumoniae* were extracted from MiBa and the pneumococcal database at SSI. For each individual, laboratory detection of the same virus infection or pneumococcal infection within 28 days was considered the same infection, and only the earliest infection was considered for this study.

We used deterministic linkage to link data from DNPR to MiBa data and the pneumococcal laboratory data through the unique personal identification number. Only individuals aged 40 years and above and who were diagnosed with both an event (myocardial infarction or stroke) and with at least one of the respiratory infections during the study period were included in the study. Vital status from the CRS registry was linked to the data to identify individuals who died at any point during the study period. Individuals could enter the study on 1 January 2010 or when they reached 40 years of age after that date and exit the study at death or at 31 December 2016. The 40 year lower age limit for inclusion in the study was chosen for comparability to other studies using a similar approach [6,7].

### Statistical analysis

We investigated the relative incidence of a first myocardial infarction or stroke occurring within 28 days after the beginning of laboratory-confirmed respiratory infections with *S. pneumoniae*, human metapneumovirus, influenza virus, parainfluenza virus, respiratory syncytial virus, or rhinovirus as compared with the baseline time period for each individual using conditional Poisson regression.

The time dependent variables such as age and season are not accounted for in the basic SCCS method, but can be included in the model [11]. Age was controlled for in 5-year bands (40–44, 45–49, 50–54, 55–59, 60–64, 65–69, 70–74, 75–79, 80–84, 85–89, 90–94, 95–99,100–104). Seasons were categorised as 3-month bands, repeated to span the study period (Jan–Mar, Apr–Jun, Jul–Sep, Oct–Dec).

Individuals experiencing acute myocardial infarction or stroke have an increased risk of dying. In the SCCS method, experiencing the outcome event should not censor follow-up, but this assumption can be violated for fatal cardiovascular events, which may lead to bias in either direction [12]. We therefore performed a sensitivity analysis, where individuals who died within 30 days following an acute myocardial infarction or stroke were excluded from the analysis to investigate this potential bias.

To investigate the potential effect modification of age on the association between respiratory infections and myocardial infarction/stroke, age was stratified on 40–64 years and ≥ 65 years [7].

Data extraction and linking of data were performed using SAS version 9.4 [15]. STATA version 13 [16] was used for the SCCS analyses.

### Ethical statement

No ethical approval was required for this register-based study

## Results

**Figure 2:**
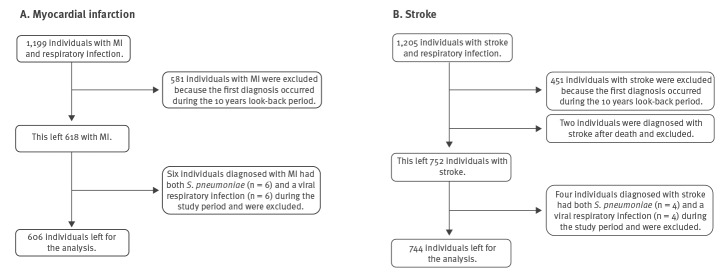
Individuals included in the study, Denmark, 1 January 2010–31 December 2016 (n = 1,350 individuals)

From 2000 to 2016, a total of 1,199 individuals with acute myocardial infarction and 1,205 individuals with stroke who also were diagnosed with either a *S. pneumoniae* or a viral respiratory infection were identified. Among these respectively, 581 with acute myocardial infarction (48%) and 451 individuals with stroke (37%), were excluded because their first acute myocardial infarction/stroke diagnosis occurred during the 10 year look-back period. This left 618 with acute myocardial infarction and 754 individuals with stroke who also had either a *S. pneumoniae* or a viral respiratory infection. When combining information on bacterial and viral respiratory infections, six individuals diagnosed with acute myocardial infarction and four diagnosed with stroke had both *S. pneumoniae* and a viral respiratory infection during the study period. Because the current study aimed to estimate the relative incidence of acute myocardial infarction and stroke separately for bacterial and viral infections, these individuals were excluded. Moreover, because the records with the two types of pathogens appeared on two different data files (i.e. bacterial or viral), these patients had each been counted twice among the respective 618 and 754 individuals with acute myocardial infection or stroke; this was accounted for when removing the records. In addition, two individuals were diagnosed with stroke after the date of death and excluded from the analysis. This left 606 individuals for the acute myocardial infarction analysis and 744 individuals for the stroke analysis (Figure 2 and Table 1).

MI: myocardial infection; *S. pneumoniae*: *Streptococcus pneumoniae*.

Ten individuals, six with MI and four with stroke, had a dual viral-bacterial infection. Each of them appeared on the two data files with viral and bacterial pathogens simultaneously, so their numbers were excluded twice.

**Table 1:** Descriptive statistics of individuals ≥ 40 years old with first myocardial infarction or stroke and at least one respiratory infection, Denmark, 1 January 2010–31 December 2016 (n = 1,350)

Characteristics	Myocardial infarction		Stroke	
	n	%	n	%
Sex				
Female	253	41.7	347	46.6
Age group in years at first cardiovascular event				
40–49	40	6.6	35	4.7
50–59	95	15.7	91	12.2
60–69	156	25.7	211	28.4
70–79	175	28.9	216	29.0
80–89	121	20.0	152	20.4
≥ 90	19	3.1	39	5.2
Total	606	100	744	100
Stratified age in years at first cardiovascular event				
40–64	210	34.7	214	28.8
≥ 65	396	65.3	530	71.2
Total	606	100	744	100
Mortality				
Mortality ≤ 30 days after cardiovascular event	51	8.4	88	11.8
Mortality during study period	242	39.9	336	45.2
Total	606	100	744	100
Laboratory-confirmed respiratory infection episodes^a,b^				
*Streptococcus pneumoniae*	222	35.4	332	43.3
Respiratory virus^c^	406	64.6	434	56.7
Total	628	100	766	100

^a^ Myocardial infarction individuals and multiple samples: one individual has four viral samples, one individual has three viral samples and 12 individuals have two viral samples. Five individuals have two bacterial samples.

^b^ Stroke individuals and multiple samples: three individuals have three viral samples, six individuals have two viral samples. One individual has three bacterial samples and eight individuals have two bacterial samples.

^c^ Human metapneumovirus, influenza virus, parainfluenza virus, respiratory syncytial virus, or rhinovirus.

Among the 606 individuals diagnosed with acute myocardial infarction, 628 respiratory infection episodes were detected. Of these, 406 infections were due to respiratory viruses with an average of 1.04 episodes per individual (range: 1–4 per individual), and 222 infections were due to *S. pneumoniae* with an average of 1.02 episodes per individual (range: 1–2 per individual) (Table 1). 

Among the 744 individuals diagnosed with stroke, a total of 766 laboratory-confirmed respiratory infections were detected during the study period. Of the 766 corresponding samples, 434 samples contained respiratory viruses with an average of 1.03 episodes per individual (range: 1–3 per individual), while 332 samples contained *S. pneumoniae* with an average of 1.03 episodes per individual (range: 1–3 per individual) (Table 1).

All in all, among individuals with acute myocardial infarction the proportion of women was 41.7%, while among individuals with stroke this was 46.6%. Individuals diagnosed with acute myocardial infarction appeared to be younger with 34.7% (210/606) aged 40–64 years compared with 28.8% (214/744) for those with stroke. The observed mortality in this study, overall, and within 30 days post-cardiovascular event, seemed highest among stroke individuals 45.2% and 11.8%, respectively, as compared with acute myocardial infarction individuals 39.9% and 8.4%, respectively (Table 1).

In the age and season adjusted analyses for first acute myocardial infarction, the IRs were significantly elevated 1–14 days following infections with *S. pneumoniae* and respiratory viruses as compared with the baseline period (Table 2). The IRs for acute myocardial infarction following an *S. pneumoniae* infection were 20.1, 11.0 and 4.9 during 1–3, 4–7 and 8–14 days, respectively. The IRs for acute myocardial infarction following a respiratory virus infection were 15.2, 4.5 and 4.4 during 1–3, 4–7 and 8–14 days, respectively.

**Table 2:** Age and season adjusted incidence ratio for first acute myocardial infarction (n = 606 individuals) and first stroke (n = 744 individuals) in periods after *Streptococcus pneumoniae,* respiratory viruses (combined) and influenza infections compared with baseline time, Denmark, 1 January 2010–31 December 2016

Days after sample collection^a^	*Streptococcus pneumoniae*			Respiratory viruses^b^ (combined)			Influenza		
	Events^c^ (n)	**IR (CI 95%)**	**p value**	**Events^c^** **(n)**	**IR (CI 95%)**	**p value**	**Events^c^** **(n)**	**IR (CI 95%)**	**p value**
Myocardial infarction									
1–3	6	20.1 (8.9–45.8)	< 0.001	8	15.2 (7.5–31.1)	< 0.001	8	17.5 (8.5–36.2)	< 0.001
4–7	4	11.0 (4.1–29.8)	< 0.001	3	4.5 (1.4–14.0)	0.010	3	5.1 (1.6–16.3)	0.005
8–14	3	4.9 (1.6–15.6)	0.006	5	4.4 (1.8–10.8)	0.001	3	3.1 (1.0–9.7)	0.056
15–28	0	ND	ND	4	1.9 (0.7–5.1)	0.205	3	1.7 (0.5–5.3)	0.374
Baseline	Ref	1 (ref)	NA	Ref	1 (ref)	NA	Ref	1 (ref)	NA
Stroke									
1–3	12	25.5 (14.2–45.8)	< 0.001	5	8.3 (3.4–20.2)	< 0.001	5	10.3 (4.2–25.4)	< 0.001
4–7	2	3.5 (0.9–14.1)	0.079	6	7.8 (3.4–17.5)	< 0.001	4	6.5 (2.4–17.7)	< 0.001
8–14	6	6.3 (2.8–14.4)	< 0.001	8	6.2 (3.0–12.6)	< 0.001	6	5.9 (2.6–13.4)	< 0.001
15–28	16	9.2 (5.5–15.4)	< 0.001	5	2.0 (0.8–4.9)	0.119	2	1.0 (0.3–4.2)	0.956
Baseline	Ref	1 (ref)	NA	Ref	1 (ref)	NA	Ref	1 (ref)	NA

CI: confidence interval; IR: incidence ratio; NA: not applicable; ND: could not be estimated (not determined); ref: reference.

^a^ The sample collection time approximates the time of symptom onset of the respiratory infection.

^b^ Human metapneumovirus, influenza virus, parainfluenza virus, respiratory syncytial virus and rhinovirus.

^c^ Events of first acute myocardial infarction or stroke.

In the age and season adjusted analyses for first stroke, the IRs of a stroke occurring during the risk period were significantly elevated 1–28 days following infection with *S. pneumoniae* except for the time period 4–7 days. The IRs for stroke following an *S. pneumoniae* infection were 25.5, 3.5, 6.3 and 9.2 during 1–3, 4–7, 8–14 and 15–28 days, respectively. The IRs for stroke following a respiratory virus infection were significantly elevated 1–14 days and the IRs were 8.3, 7.8 and 6.2 during 1–3, 4–7 and 8–14 days, respectively (Table 2).

In the age and season adjusted sub-analyses for first acute myocardial infarction and stroke where only influenza virus infections were included, the estimated IRs were similar to the IRs obtained in the analysis including all respiratory virus infections (Table 2).

### Excluding those who died within 30 days

In total 51 (8.4%) and 88 (11.8%) individuals died within 30 days after acute myocardial infarction and stroke, respectively (Table 1). Excluding those who died within 30 days after an event provided results that were mostly unchanged from the main analysis (Table 3), although with slightly attenuated effect estimates. Using the risk period 1–3 days as an example, the acute myocardial infarction IR after *S. pneumoniae* infection changed from 20.1 to 18.2 and for respiratory viruses the IR changed from 15.2 to 14.8. The stroke IR after *S. pneumoniae* infection changed from 25.5 to 21.9 and for respiratory virus the change was from 8.3 to 7.9.

**Table 3:** Age and season adjusted incidence ratio for first acute myocardial infarction (n = 555) and first stroke (n = 656) after excluding patients who died within 30 days of S*treptococcus pneumoniae* and respiratory viruses infections (combined) compared with baseline time, Denmark, 1 January 2010–31 December 2016

Days after sample collection^a^	*Streptococcus pneumoniae*		Respiratory viruses^b^ (combined)	
	IR (CI 95%)	p value	IR (CI 95%)	p value
Myocardial infarction				
1–3	18.2 (7.4–44.6)	< 0.001	14.8 (6.9–31.6)	< 0.001
4–7	11.4 (4.2–30.9)	< 0.001	4.9 (1.6–15.4)	0.006
8–14	3.4 (0.8–13.6)	0.090	3.9 (1.4–10.4)	0.008
15–28	ND	ND	2.0 (0.8–5.5)	0.157
Baseline	1 (ref)	NA	1 (ref)	NA
Stroke				
1–3	21.9 (10.7–44.6)	< 0.001	7.9 (2.9–21.2)	< 0.001
4–7	2.2 (0.3–15.9)	0.425	7.6 (3.1–18.5)	< 0.001
8–14	7.8 (3.5–17.7)	< 0.001	6.2 (2.9–13.3)	< 0.001
15–28	9.6 (5.5–16.6)	< 0.001	1.4 (0.4–4.4)	0.564
Baseline	1 (ref)	NA	1 (ref)	NA

CI: confidence interval; IR: incidence ratio; NA: not applicable; ND: could not be estimated; ref: reference.

^a^ The sample collection time approximates the time of symptom onset due to infection with either *Streptococcus pneumoniae *or respiratory viruses^.^

^b^ Human metapneumovirus, influenza virus, parainfluenza virus, respiratory syncytial virus and rhinovirus.

### Stratifying by age

For both acute myocardial infarction and stroke, the number of Individuals aged between 40 and 64 years of age covered approximately one third of the total number of individuals included in the main analyses, for some risk periods no events were observed and IR could not be estimated. In addition, several IR estimates had wide confidence intervals. In particular the estimated IRs for the 1–3 days risk periods appeared to be higher in the 40–64 years age stratified analyses compared with what we observed in the main analysis (Table 2). Following onset of an *S. pneumoniae* infection, the acute myocardial infarction IR was 37.1 and for stroke the IR was 43.3 during the 1–3 days risk period among 40–64 year olds. In the 1–3 days after onset of a respiratory virus infection, the acute myocardial infarction IR was 5.1 and non-significant while during the same period the stroke IR was 16.4 and significant (Table 4). IRs for all risk periods are shown in Table 4. For the age group ≥ 65 years the acute myocardial infarction and stroke IRs were similar to the IR estimates observed in the main analyses. Acute myocardial infarction and stroke IRs for all risk periods are shown in Table 4.

**Table 4:** Age and season adjusted incidence ratio for first acute myocardial infarction (n = 606) and first stroke (n = 744) stratified by age 40–64 years and ≥ 65 years in periods after *Streptococcus pneumoniae* and respiratory viruses infections (combined) compared with baseline time, Denmark, 1 January 2010–31 December 2016

Days after sample collection^a^	*Streptococcus pneumoniae*		Respiratory viruses^b^ (combined)	
	IR (CI 95%)	p value	IR (CI 95%)	p value
Myocardial infarction				
40–64 years (n = 210)				
1–3	37.1 (8.7–158.3)	< 0.001	5.1 (0.7–36.6)	0.108
4–7	14.6 (2.0–108.7)	0.009	7.6 (1.9–31.2)	0.005
8–14	ND	ND	2.2 (0.3–16.1)	0.426
15–28	ND	ND	1.2 (0.2–8.4)	0.880
Baseline	1 (ref)	NA	1 (ref)	NA
≥ 65 years (n = 396)				
1–3	16.1 (5.5–47.2)	< 0.001	21.1 (9.8–45.6)	< 0.001
4–7	10.7 (3.2–35.0)	< 0.001	2.4 (0.3–17.3)	0.381
8–14	6.2 (1.9–20.5)	0.003	5.9 (2.1–16.0)	0.001
15–28	ND	ND	2.4 (0.8–7.6)	0.134
Baseline	1 (ref)	NA	1 (ref)	NA
Stroke				
40–64 years (n = 214)				
1–3	43.3 (15.3–122.5)	< 0.001	16.4 (5.1–52.89)	< 0.001
4–7	NA	NA	8.4 (2.0–34.7)	0.003
8–14	10.3 (2.5–43.2)	0.001	10.4 (3.7–28.9)	< 0.001
15–28	14.1 (5.5–36.1)	< 0.001	ND	ND
Baseline	1 (ref)	NA	1 (ref)	NA
≥ 65 years (n = 530)				
1–3	20.6 (10.1–42.3)	< 0.001	4.9 (1.2–20.0)	0.025
4–7	4.3 (1.1–17.4)	0.041	7.7 (2.9–21.0)	< 0.001
8–14	5.3 (1.9–14.2)	0.001	4.6 (1.7–12.5)	0.003
15–28	7.9 (4.2–14.7)	< 0.001	3.0 (1.2–7.4)	0.016
Baseline	1 (ref)	NA	1 (ref)	NA

CI: confidence interval; IR: incidence ratio; NA: not applicable; ND: could not be estimated (no events); ref: reference.

^a^ The sample collection time approximates the time of symptom onset due to infection with either *Streptococcus pneumoniae *or respiratory viruses^.^

^b^ Human metapneumovirus, influenza virus, parainfluenza virus, respiratory syncytial virus and rhinovirus.

## Discussion

We found that the IRs of first acute myocardial infarction and stroke were markedly elevated following infections with *S. pneumoniae* and selected respiratory viruses (mainly influenza virus) compared with baseline time periods using national linked records from Denmark. The elevated IRs were observed during the risk periods 1–14 days following infection, except for the stroke IRs following *S. pneumoniae* infection where an elevated IR was observed during the whole risk period apart from the 4–7 day period.

The IR patterns observed during the risk periods in our study were similar to the patterns observed in the Scottish and Canadian studies with the highest IRs observed 1–3 days after infection followed by a decrease in IRs with increasing time since infection [5,7]. The estimated IRs were in general higher in our study compared with the Scottish and Canadian studies in particular during the risk window 1–3 days following infection. The IRs for acute myocardial infarction following *S. pneumoniae* and respiratory virus infections in the Scottish study were 5.98 and 5.59, respectively 1–3 days following infection, compared with 20.1 and 15.2 in our study. The IRs for acute myocardial infarction 1–3 days following influenza infection were 9.8 in the Scottish study [7], 6.3 in the Canadian study [5] and 17.5 in our study. The IR for stroke in the Scottish study was 12.3 following *S. pneumoniae* infection and 6.79 following infection with respiratory viruses during the 1–3 days risk window. In our study, the corresponding IRs were 25.5 and 8.3. In the Scottish study, acute myocardial infarction and stroke IRs were higher following influenza infections compared with other respiratory viruses. In our study, the sample size of other viruses than influenza only accounted for 20% of all respiratory viruses; therefore, due to the small sample size, it was not possible to make a similar comparison based on Danish data.

In the Scottish study, the estimated IRs for the effect of respiratory viruses were generally higher among individuals aged 40–64 years compared with those aged ≥ 65 years, and this was suggested to possibly be due to a higher influenza vaccine coverage among those aged ≥ 65 years [7]. In our study, the IRs associated with respiratory viruses were not in general higher among individuals below 65 years old, although confidence intervals were wide. However, the IRs for myocardial infarction and stroke 1–3 days following *S. pneumoniae* infection in the age group 40–64 years were more than double those of the age group ≥ 65 years. Based on the assumption that the pneumococcal vaccine may reduce the risk of cardiac outcomes, either by preventing infection or lessening its severity, this might indicate that individuals aged ≥ 65 years are aware of getting their recommended pneumococcal vaccine. A similar pattern was observed for stroke in the Scottish study [7]. However, the differences between the age strata observed in the Scottish study and ours require further investigation and it would be interesting to repeat the analysis with a larger sample size stratifying on influenza and *S. pneumoniae* vaccination status. Additionally analyses with stratifications based on influenza and pneumococcal subtypes, as well as taking into account the underlying comorbidities individuals may have, may provide knowledge allowing to further customise/target the influenza and pneumococcal vaccination programmes.

Acute myocardial infarction and stroke are related to a high mortality rate, which can result in a shortening of the observation period leading to bias in either direction [12]. In both the Scottish [7] and in our study, the estimated IRs when excluding those who died within 30 days after the event produced results similar to the main analysis indicating that the high mortality did not bias the results (Table 2 and 3).

Individuals ≥ 65 years and people at any age diagnosed with cardiovascular risk factors where an influenza infection can result in severe health effects can get the influenza vaccine for free in Denmark. However, they might not be aware that they have increased risk of acute myocardial infarction and stroke following an influenza infection. Genetic changes or differences in the circulating influenza viruses compared with the vaccine strains in some seasons can result in low or suboptimal vaccine effectiveness [17], however, in these seasons improvement of influenza vaccine coverage will still increase number of prevented influenza cases. *S. pneumoniae* is also vaccine preventable and the vaccine is recommended to individuals ≥ 65 years, but in Denmark only individuals with very high risk of invasive pneumococcal disease (IPD) are covered by limited subsidy when vaccinated [18]. However there are other groups with increased risk of IPD where pneumococcal vaccination is recommended but not free of charge e.g. all individuals ≥ 65 years of age who do not have an IPD risk classified as very high [19,20]; individuals of all ages diagnosed with chronic conditions such as heart disease and lung disease [21,22].

Strengths of our study include (i) use of SCCS analysis which implicitly controls for fixed between-person confounding effects and is statistically efficient compared with a cohort design, (ii) use of laboratory-confirmed infections from a national microbiology database combined with ICD-10 coded cardiovascular outcomes, which are shown to have high positive predictive values in validation studies from Denmark [23-25] that enhance confidence in the validity of measurements, (iii) use of national linked data across all of Denmark and (iv) use of the same protocol as the Scottish study to allow for direct comparisons between the studies. Although our study period was shorter and the sample size smaller than the Scottish one, the main results obtained by the two studies are comparable.

Our study also has limitations. With a sample size of 606 individuals in the acute myocardial infarction analyses, and 744 in the stroke analyses, totalling 1,350 individuals our study was smaller than the Scottish study [7], which resulted in lack of power in some sub-group analyses. In addition, there were too few non-influenza respiratory viruses to separate the respiratory virus analyses according to influenza vs non-influenza viruses, and the limited sample size and the short duration of the time series did not enable us to disentangle influenza subtypes effects. Only very few individuals experienced co-infections and the effect of co-infections could not be investigated separately. For the respiratory infections we used date of sampling and not date of symptom onset, which means that the true date of infection occurred during the 14 days excluded from the analyses. The IRs decreased as time since sampling increased. The fact that we use date of sampling, which is after the date of infection, may lead to underestimation of the effect. The decision to swab an individual to verify a respiratory infection might be more likely for individuals with a severe infection or underlying risk factors for severe disease, which might indicate that the results from this study apply to more severe infections and/or vulnerable patients [5].

In conclusion, this study suggested a significant cardiovascular event triggering effect following infection with *S. pneumoniae* and respiratory viruses (mainly influenza). This was found not only in the age group ≥ 65 years, but also among 40–64 year olds. The results were similar to those found in a Scottish population using the same study protocol, suggesting that they are generalisable across different northern European populations. These findings indicate the importance to inform general practitioners and individuals at risk of cardiovascular diseases about the importance of being protected against vaccine-preventable respiratory infections.
